# *miR-34a *and *miR-15a/16 *are co-regulated in non-small cell lung cancer and control cell cycle progression in a synergistic and Rb-dependent manner

**DOI:** 10.1186/1476-4598-10-55

**Published:** 2011-05-16

**Authors:** Nora Bandi, Erik Vassella

**Affiliations:** 1 Institute of Pathology, University of Bern, Bern, Switzerland

**Keywords:** cell cycle control, microRNA, non-small cell lung cancer, retinoblastoma, synergism

## Abstract

**Background:**

microRNAs (miRNAs) are small non-coding RNAs that are frequently involved in carcinogenesis. Although many miRNAs form part of integrated networks, little information is available how they interact with each other to control cellular processes. *miR-34a *and *miR-15a/16 *are functionally related; they share common targets and control similar processes including G_1_-S cell cycle progression and apoptosis. The aim of this study was to investigate the combined action of *miR-34a *and *miR-15a/16 *in non-small cell lung cancer (NSCLC) cells.

**Methods:**

NSCLC cells were transfected with *miR-34a *and *miR-15a/16 *mimics and analysed for cell cycle arrest and apoptosis by flow cytometry. Expression of retinoblastoma and cyclin E1 was manipulated to investigate the role of these proteins in miRNA-induced cell cycle arrest. Expression of miRNA targets was assessed by real-time PCR. To investigate if both miRNAs are co-regulated in NSCLC cells, tumour tissue and matched normal lung tissue from 23 patients were collected by laser capture microdissection and compared for the expression of these miRNAs by real-time PCR.

**Results:**

In the present study, we demonstrate that *miR-34a *and *miR-15a/16 *act synergistically to induce cell cycle arrest in a Rb-dependent manner. In contrast, no synergistic effect of these miRNAs was observed for apoptosis. The synergistic action on cell cycle arrest was not due to a more efficient down-regulation of targets common to both miRNAs. However, the synergistic effect was abrogated in cells in which cyclin E1, a target unique to *miR-15a/16*, was silenced by RNA interference. Thus, the synergistic effect was due to the fact that in concerted action both miRNAs are able to down-regulate more targets involved in cell cycle control than each miRNA alone. Both miRNAs were significantly co-regulated in adenocarcinomas of the lung suggesting a functional link between these miRNAs.

**Conclusions:**

In concerted action miRNAs are able to potentiate their impact on G_1_-S progression. Thus the combination of miRNAs of the same network rather than individual miRNAs should be considered for assessing a biological response. Since miR-34a and miR-15a/16 are frequently down-regulated in the same tumour tissue, administrating a combination of both miRNAs may also potentiate their therapeutic impact.

## Background

Lung cancer is the leading cause of cancer-related death in industrialized countries [1]. Systemic treatment of lung cancer patients includes chemotherapy, inhibitors of angiogenesis and inhibitors of EGFR signaling. However, since the effect of these drugs is only transient, the overall five-year survival rate is less than 15%. Non-small cell lung carcinoma (NSCLC) accounts for 80% of lung cancer and is further subdivided into two major types, squamous cell carcinoma and adenocarcinoma [2]. Squamous cell carcinoma usually arises from the major bronchi, whereas adenocarcinoma arises from distant airway bronchioles and alveoli. These tumours show frequent alterations of genes involved in cell cycle control or apoptosis including *k-RAS*, *EGFR*, *c-Myc*, *cyclin D1 *(*CCND1*), *TP53*, *retinoblastoma *(*Rb)*, *p16INK *and *Bcl2 *[3], but the relevant molecular mechanisms driving the aggressive biological behaviour of these tumours are largely unknown.

miRNAs are small regulatory RNA molecules at the post-transcriptional level and are implicated in a wide variety of biological processes including proliferation, differentiation and apoptosis [4]. Notably, miRNAs form networks to regulate the expression of individual components of the cell cycle control machinery. Many of these miRNAs including the *let-7 *family [5], *miR-34 *[6], *miR-15a/16 *[7], *miR-221/222 *[8,9], *miR-17-92 *[10], *miR-107 *and *miR-185 *[11] are frequently dysregulated in lung cancer and therefore constitute promising targets for specific anticancer intervention (reviewed by Negrini et al. [12]).

Many miRNAs are implicated in cell cycle progression or apoptosis, but surprisingly little information is available if these miRNAs are able to interact with each other to co-ordinately regulate these cellular processes. In addition, it is poorly understood why miRNAs often share common targets despite the fact that they constitute a relatively small family of RNAs encoded by less than 1000 genes. In this study we have analysed two miRNAs, *miR-15a/16 *and *miR-34*, which are located at chromosomal regions 13q14 and 1p34, respectively. Although these miRNAs contain completely unrelated seed sequences, they are functionally related since they are both able to induce G_1_-G_0 _cell cycle arrest and apoptosis [7,13-15]. In addition, they share common targets including *CCND1*, *CDK4*, *CDK6*, *E2F3 *and *Bcl2*. However, other targets also exist which are unique to *miR-15a/16 *(c*yclin E1 *(*CCNE1)*, *cyclin D2 *(*CCND2*) or *cyclin D3 *(*CCND3*)) or *miR-34a *(*c-Myc*, *n-Myc*, and *c-Met*) [7,16-18].

To investigate if these miRNAs are able to interact with each other for the regulation of cellular processes, they were overexpressed in NSCLC cell lines. Here we demonstrate that *miR-15a/16 *and *miR-34 *act synergistically to induce cell cycle arrest in a Rb-dependent manner. The synergistic effect can be explained by the fact that in concerted action, miRNAs are able to down-regulate more targets than each miRNA alone. Thus, it may be important to analyse miRNAs in a combinatorial mode as this may provide additional information on their role in specific cellular processes. Consistent with these findings, both miRNAs are frequently down-regulated in adenocarcinomas and squamous cell carcinomas of the lung. Our results suggest that targeting a combination of miRNAs involved in the same pathway may potentiate the therapeutic effect of each individual miRNA.

## Materials and methods

### Cell lines and culture conditions

The NSCLC cell lines A549, H2009, H1299 and H358 were obtained from the American Type Culture Collection, Rockville, MD. All cell lines were cultured in Iscove's modified Dulbecco's medium supplemented with 2 mM L-alanyl-L-glutamine, 1% penicillin/streptomycin and 5% foetal bovine serum (Sigma) at 37°C and 5% CO_2_.

### Transfection

Cells were seeded in culture flasks 24 h prior to transfection. Co-transfections with plasmid DNA were performed using Effectene reagent (Qiagen), all other transfections were performed using HiPerFect (Qiagen). If not otherwise specified, transfection was performed using 20 nM of hsa-*pre-miR-34a*, a mixture of 10 nM hsa-*pre-miR-15a *and 10 nM hsa-*pre-miR-16 *or 20 nM pre-miR miRNA precursor control 1 (Ambion). Si RNAs against Rb, or CCNE1 (siGENOME SMARTpool, Dharmacon) were used at 60 nM or 7.8 nM, respectively. Control transfections were performed using non-targeting Pool 2 (siGENOME). pCMV-Rb [19] or empty control plasmid were used at 125 ng/ml. Transfection efficiency of short RNAs and plasmid DNA was monitored using siGloGreen transfection indicator (Dharmacon) or an RFP-expression plasmid, respectively.

### Cell cycle analysis and cell death assay

Cell cycle analysis was performed by flow cytometry essentially as described [7]. For cell death analysis, floating and adherent cells were harvested, combined, washed with PBS and stained with 10 μg/ml propidium idode (Sigma). Apoptotic cells were detected using an antibody directed against cleaved caspase 3 (clone 5A1E, 1:100, Cell Signaling) as described [20]. Cells were analysed using an LSR II flow cytometer (BD Biosciences) and FlowJo 8.8.4 software (Tree Star). As a positive control, cells were treated with UV (400 mJ) using a UV Stratalinker 1800 (Stratagene).

### RNA isolation and real-time PCR

Total RNA was extracted from cultured cells using the miRVana RNA isolation kit according to the manufacturer's instructions (Ambion). TaqMan miRNA assays (Applied Biosystems) were performed as described [7] using a Real-Time PCR system 7500 (Applied Biosystems). miRNA levels were normalized to the level obtained for *RNU48*. Quantification of *Bcl2 *was done using a TaqMan assay (Applied Biosystems); all other mRNAs were quantified using Quantitec primer assays (Qiagen). mRNA levels were normalized to the level obtained for *GAPDH*. Changes in expression were calculated using the ΔΔCt method.

### Western blot analysis

Western blot analysis was performed as described [7]. Monoclonal antibodies against Rb (clone 3C8, QED Bioscience) and phospho-Rb (Ser807/811, Cell Signaling) were diluted 1:1000, monoclonal antibody against Bcl2 (clone 124, Dako) was diluted 1:300, and monoclonal antibody against α-tubulin (clone B512, Sigma) was diluted 1:5000. Secondary goat anti-mouse-HRP and goat anti-rabbit-HRP antibodies (Biorad) were used at 1:5000 or 1:7000, respectively.

### Laser capture microdissection

Formalin-fixed paraffin-embedded tissues from 11 adenocarcinomas and 12 squamous cell carcinomas were used for miRNA expression analysis. Tumour tissues and corresponding normal tissues from bronchiolar or alveolar epithelium, respectively, was collected by laser capture microdissection as described previously [7]. Stroma components including connective tissues, inflammatory cells and blood vessels were excluded. Nucleic acids were subjected to a heat-treatment in order to remove methylol groups introduced during formalin fixation and subjected to real-time PCR as described [7]. All experiments using human specimens were done according to the ethical guidelines of the Institute of Pathology, University of Bern, and were reviewed by the institutional review board.

### Statistics

Statistical analyses were performed using the GraphPAD prism software. Statistical differences were calculated using unpaired two-tailed student's t-test. A probability of p ≤ 0.05 was considered statistically significant. Statistical significance of correlation was assessed by the Pearson test.

## Results

### *miR-15a/16 *and *miR-34a *are co-regulated in adenocarcinomas of the lung

**Figure 1:**
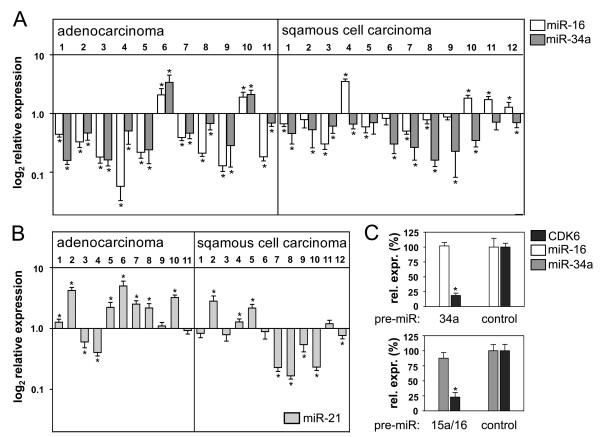
***miR-34a *and *miR-15a/16 *are co-regulated in non-small cell lung cancer**. (A) miR-16 and *miR-34a *levels in adenocarcinomas and squamous cell carcinomas relative to matched normal tissues. (B) *miR-21 *levels in the same tumour samples, relative to matched normal tissue. (C) *miR-34a *and *miR-15a/16 *are not able to mutually regulate their expression. H2009 cells were transfected with *pre-miR-34a *(upper panel) or *pre-miR-15a/16 *(lower panel) and analysed for the expression of the miRNA counterpart or *CDK6 *mRNA 42 h post-transfection. Values are relative to the value obtained for the control transfected with precursor control (n = 3). *, P < 0.05.

The finding that *miR-15a/16 *and *miR-34a *share many common targets involved in G_1 _progression prompted us to analyse if these miRNAs are co-regulated in NSCLC. Tumour tissues of adenocarcinomas and squamous cell carcinomas and matched normal tissues from alveolar or bronchial epithelium, respectively, were collected by laser capture microdissection and compared for the expression of both miRNAs by real-time PCR. We have shown previously that *miR-15a *and *miR-16 *are frequently down-regulated in NSCLC [7]. Here we demonstrate that the expression of *miR-16 *is significantly correlated to the expression of *miR-34a *in adenocarcinomas (p = 0.018), but not in squamous cell carcinomas of the lung. Both miRNAs were down-regulated in 82% (9/11), and up-regulated in 18% (2/11) of adenocarcinomas (Figure 1A). In contrast, *miR-34a *was significantly down-regulated in 10/12 squamous cell carcinoma samples while *miR-16 *was down-regulated in 5/12 tumour samples. Both miRNAs were also significantly down-regulated in the NSCLC cell lines A549, H2009, H1299 and H358 (data not shown).

Co-repression of miRNAs may be due to defects in miRNA processing. Notably, reduced expression of Dicer has been detected in NSCLC [21]. To address this possibility, the same tumour tissues were analysed for the expression of *miR-21*, which is frequently up-regulated in lung cancer [22,23]. As shown in Figure 1B, *miR-21 *was up-regulated or expressed at normal levels in 9 of 11 adenocarcinomas and 7 of 12 squamous cell carcinomas. Thus, abrogation of miRNA processing may only account for a small subgroup of NSCLC samples.

We next investigated the possibility that both miRNAs are linked because they are able to mutually regulate their expression. To this end, *miR-15a/16 *or *miR-34a *were overexpressed by transfection with miRNA precursors (pre-miRNA) in a Rb-deficient NSCLC cell line, H2009, and analysed for the expression of the miRNA counterpart. Since H2009 is refractory to cell cycle arrest induced by *miR-15a/16 *[7] and *miR-34a *(see below), secondary effects on miRNA expression as a consequence of the G_1_-G_0 _arrest can be excluded using this cell line. However, neither *miR-15a/16 *nor *miR-34a *was able to affect the expression of its counterpart, while *CDK6 *mRNA, a target common to both miRNAs, was significantly down-regulated (Figure 1C).

### *miR-34a*-induced cell cycle arrest depends on the expression of Rb

One miRNA can affect the expression of hundreds of proteins [24], which often renders it difficult to identify the relevant targets. We have previously shown that *miR-15a/16*-induced cell cycle arrest depends on the expression of Rb. This indicates that components of the cell cycle machinery upstream of Rb, including *cyclin D1 *(*CCND1*), *cyclin D3 *(*CCND3*), *CDK4 *and *CDK6 *[7,25,26] , are the most relevant targets of *miR-15a/16*. To investigate if *miR15a/16 *and *miR-34a *share redundant functions, *miR-34a *was analysed using the same set of experiments as we have described previously for *miR-15a/16 *[7].

**Figure 2:**
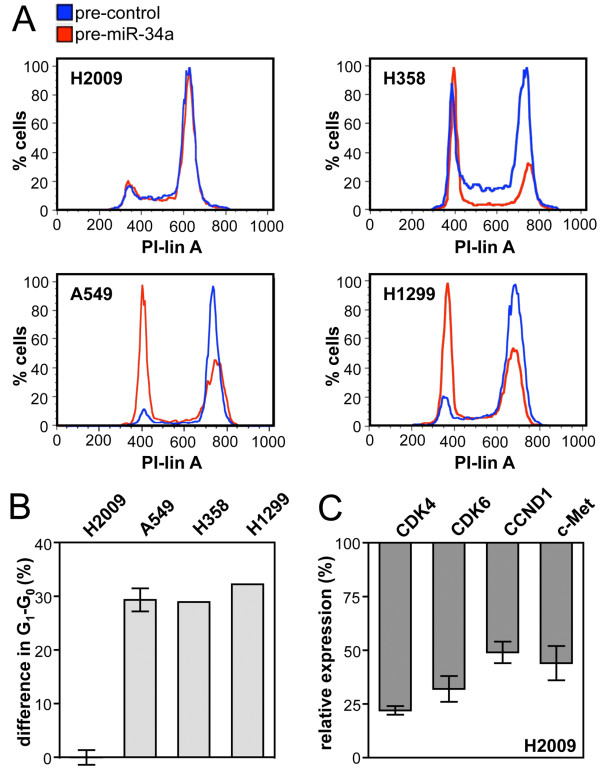
**H2009 cells are refractory to *miR-34a*-induced cell cycle arrest**. (A) DNA content distribution of NSCLC cells transfected with precursor miRNA or precursor control. Cells were treated for 18 h with nocodazole beginning 24 h post-transfection. (B) Percent difference in G_1_-G_0 _between cells transfected with *pre-miR-34a *and cells transfected with precursor control. H2009, A549, n = 3; H1299 and H358, n = 1. (C) mRNA levels of known *miR-34a *targets. H2009 cells were transfected with *pre-miR-34a *and harvested 42 h post-transfection (n = 3). Values are relative to the level obtained for the control transfected with precursor control.

**Figure 3:**
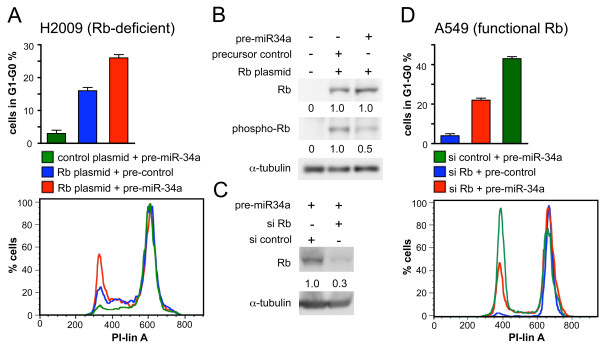
***miR-34a*-induced cell cycle arrest depends on the expression of Rb**. (A, D), DNA content distribution by flow cytometry of H2009 (A) and A549 cells (D) treated for 18 h with nocodazole beginning 48 h post-transfection. The percent cells in G_1_-G_0 _is shown in the upper panel (n = 3) and a representative histogram of the cell cycle profile is shown in the lower panel. (B, C), Western blot analysis of H2009 (B) and A549 cells (C) subjected to the same conditions as in (A) and (D) using antibodies directed against Rb or phospho-Rb. Protein levels were normalised to α-tubulin.

To investigate cell cycle arrest, the NSCLC cell lines A549, H358, H1299 and H2009 were transfected with *pre-miRNA-34a *and treated with nocodazole 24 h post-transfection. Nocodazole traps cells at the G_2_-M phase, but ~30% of the transfected A549, H358 or H1299 cells accumulated in G_1_-G_0 _(Figure 2A and 2B) indicating that *miR-34a *induces an arrest in this phase of the cell cycle. In contrast, Rb-deficient H2009 cells were completely refractory to *miR-34a*-induced arrest (Figure 2A and 2B). However, known targets of *miR-34a *including *CDK4*, *CDK6*, *CCND1 *and *c-Met *were significantly down-regulated in these cells (Figure 2C). Rb reconstitution into Rb-deficient NSCLC lines restores G_1 _arrest mechanisms [27]. To investigate if *miR-34a*-induced cell cycle arrest depends on the expression of Rb, the latter gene was reintroduced into H2009 cells. Co-transfection of H2009 cells with *pre-miR-34a *and an empty control plasmid induced cell cycle arrest in 3.1 ± 1% of the population (Figure 3A). Consistent with previous findings [27], the percentage of cells in the G_1_-G_0 _phase of the cell cycle increased to 16 ± 1% upon transfection with a Rb expression plasmid (p = 0.001). This indicates that Rb per se is able to induce cell cycle arrest in a significant proportion of the population. However, Rb plasmid in combination with *pre-miR-34a *induced cell cycle arrest in significantly more cells (26 ± 1%, p = 0.01) than Rb plasmid in combination with the precursor control. The expression of Rb protein was not affected by *pre-miR-34a*. In contrast, phospho-Rb was reduced by 50% under these conditions (Figure 3B). In conclusion, the ability of *miR-34a *to induce cell cycle arrest depends on the expression of Rb. Complementary experiments were performed in A549 cells, in which the Rb gene was knocked down by RNA interference. The knock-down expressed three times less Rb protein than the control (Figure 3C). As expected, the knock-down was significantly more resistant to *miR-34a*-induced cell cycle arrest (22 ± 1% in G_1_-G_0_) than the control (43 ± 1% in G_1_-G_0_, p < 0.001) (Figure 3D). In conclusion, there is a significant degree of redundancy between *miR-34a *and *miR-15a/16 *in their ability to induce cell cycle arrest in NSCLC cells.

### *miR-15a/16 *and *miR-34a *act synergistically to induce arrest in G_1_-G_0_

**Figure 4:**
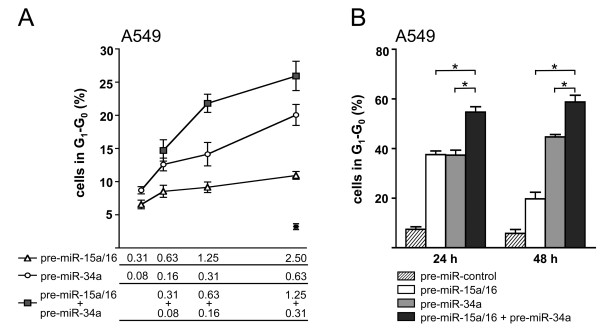
***miR-15a/16 *and *miR-34a *act synergistically to induce cell cycle arrest**. (A) Cell cycle analysis of A549 cells transfected with *pre-miR-34a *and/or *pre-miR-15a/16 *under non-saturating conditions. Precursors were supplemented with precursor control to yield a total concentration of 2.5 nM per transfection. *, transfection with 2.5 nM precursor control. Cells were treated for 18 h with nocodazole beginning 24 h post-transfection (n = 3). (B) Cell cycle analysis under saturating conditions. A549 cells were transfected with 20 nM precursor or precursor control or co-transfected with 10 nM *pre-miR-34a *and 10 nM *pre-miR-15a/16 *and treated for 18 h with nocodazole beginning 24 h (left panel) or 48 h (right panel) post-transfection (n = 3).

We next addressed the question if *miR-15a/16 *and *miR-34a *act together to induce cell cycle arrest. A549 cells were transfected with increasing concentrations of *pre-miR-15a/16 *or *pre-miR-34a *and analysed for cell cycle arrest (Figure 4A). From the slope of the dose-response curves it can be deduced that *pre-miR-34a *was more efficient than pre-*miR-15a/16 *in inducing cell cycle arrest. We next assessed the concerted action of *miR-15a/16 *and *miR-34a *precursors on cell cycle arrest. Transfection with 2.5 nM *pre-miR-15/16 *or transfection with 0.63 nM *pre-miR-34a *resulted in a G_1_-G_0 _arrest of A549 cells in 10.9 ± 0.6% and 20.1 ± 1.6% of the population, respectively. Interestingly, a mixture with half the concentrations of *pre-miR-15/16 *and *pre-miR-34a *was more efficient (25.9 ± 2.2%) than each pre-miRNA alone in inducing a G_1_-G_0 _arrest, p ≤ 0.02 (Figure 4A). Consistent results were obtained over a four-fold concentration range. Thus, these results clearly indicate that *miR-15a/16 *and *miR-34a *act synergistically to induce cell cycle arrest in G_1_-G_0_.

Both *pre-miRNAs *displayed saturation for cell cycle arrest at a concentration of 20 nM (data not shown). Interestingly, a synergistic effect was also obtained at this concentration: cells transfected with 20 nM *pre-miR-15a/16 *or 20 nM *pre-miR-34a *in each case gave rise to a G_1_-G_0 _arrest in about 37% of the population. In contrast, co-transfection of cells with both pre-miRNAs at half the concentration (10 nM each) resulted in a G_1_-G_0 _arrest in 54.6 ± 2.2% of the population (Figure 4B, 24 h), p < 0.0005. The synergistic effect was observed 24 h and 48 h post-transfection (Figure 4B).

### No synergistic action on cell death

**Figure 5:**
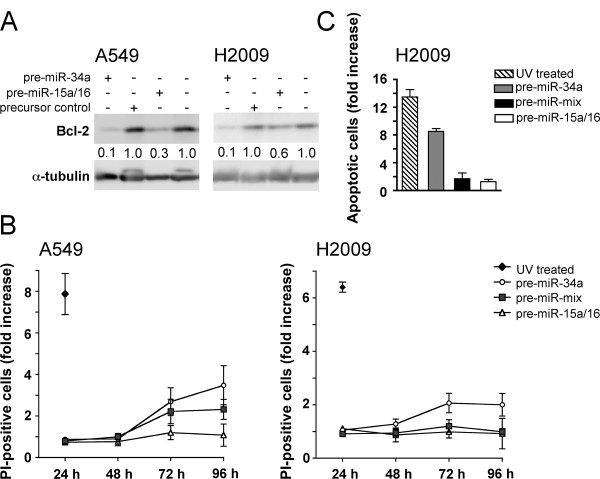
**No synergism on cell death**. (A) Bcl2 expression. A549 or H2009 cells were transfected with 20 nM precursors and harvested 42 h post-transfection. Western blot analysis was performed using a monoclonal antibody against Bcl2. Protein levels were normalized to α-tubulin and presented relative to the level obtained for the control. (B) Time-course of propidium iodide (PI)-positive A549 and H2009 cells by flow cytometry. Cells were transfected with concentrations of precursors as indicated in the legend to Fig. 4B (n = 3). Cells were gated as shown in Additional file 1A. (C) Cleaved caspase-3-positive cells. H2009 cells were analysed for the presence of cleaved caspase 3 by flow cytometry 72 h post-transfection (n = 3). Values are relative to the level obtained for the control transfected with precursor control. As a positive control, cells were treated with UV.

*miR-15a/16 *and *miR-34a *are both able to down-regulate the anti-apoptotic protein *Bcl2 *(Figure 5A). In addition, both miRNAs have been shown to induce apoptosis in different cell systems [13,15]. We therefore wondered if these miRNAs also act synergistically to induce apoptosis. In line with previous findings we were unable to detect any significant increase in propidium iodide (PI)-positive A549 or H2009 cells on transfection with *miR-15a/16 *(Figure 5B and Additional file 1). Likewise, no significant increase in cleaved caspase-3-positive cells was observed (Figure 5C). In contrast, *miR-34a *elicited a 2-3-fold increase in PI-positive cells and an eight-fold increase in cleaved caspase-3-positive cells 72-96 h post-transfection (Figure 5B and 5C). Surprisingly, a mixture of both pre-miRNAs at half the concentration was less efficient than *miR-34a *alone in inducing cell death. In conclusion, no synergism elicited by *miR-15a/16 *and *miR-34a *exists for cell death in NSCLC cells.

### Combined action of *miR-15a/16 *and *miR-34a *on the stability of individual mRNA targets

**Figure 6:**
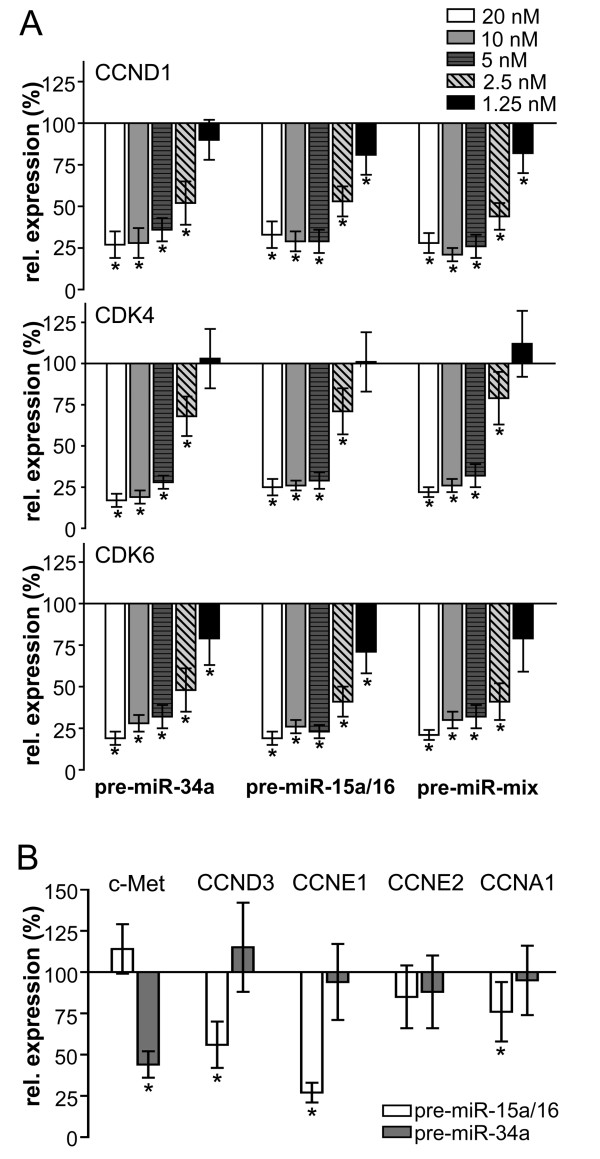
**Concerted action of *miR-15a/16 *and *miR-34a *on individual mRNA targets**. (A) mRNA levels of targets common to both miRNAs. H2009 cells were transfected with *pre-miR-15a/16 *or *pre-miR-34a *alone or co-transfected with both pre-miRNAs together (pre-miR-mix) at concentrations as indicated in the figure. (B) Expression level of targets unique to *miR-15a/16 *or *miR-34a*. Analysis was performed as described in the legend to Fig. 2C (n = 3). *, p < 0.05.

We next investigated the mechanism underlying the synergistic action of *miR-34a *and *miR-15a/16*. It is possible that the observed effect is due to a more efficient down-regulation of targets common to both miRNAs by a combined action of *miR-34a *and *miR-15a/16*. To circumvent adverse secondary effects on target gene expression of individual G_1 _proteins as a consequence of cell cycle arrest, the cell line H2009 was again used for the experiment. Transfection with serial dilutions of pre-miRNAs allowed the establishment of a direct relationship between the amount of input pre-miRNA and the steady-state level of mRNA of the target genes *CDK4, CDK6 *and *CCND1*, respectively (Figure 6A and Additional file 2).

Interestingly, transfection of H2009 cells with *miR-34a *or *miR-15a/16*, or co-transfection of H2009 cells with a mixture of both pre-miRNAs at half the concentration gave rise to comparable dose-response curves for all the three target genes (Figure 6A). From these results we may conclude that *miR-15a/16 *and *miR-34a *act in an additive rather than in a synergistic manner on individual mRNAs. Comparable results were obtained in H2009 and A549 cells excluding the possibility that the concerted action on individual mRNA targets depends on the expression of Rb (data not shown).

### The synergistic action of *miR-15a/16 *and *miR-34a *is due to the down-regulation of additional genes involved in G_1 _progression

Although *miR-15a/16 *and *miR-34a *share many common targets, other targets exist which are unique to *miR-15a/16 *or *miR-34a *(Additional file 2). Since no synergistic effect was observed for individual mRNA targets, we hypothesized that the observed effect may be due to the fact that more targets involved in G_1_-S progression are repressed by the combined action of both miRNAs. Target specificity was re-evaluated using the cell line H2009, which is refractory to miRNA-induced arrest. In agreement with reports from the literature [7,16], down-regulation of *c-Met *mRNA was specific for *miR-34a*, whereas down-regulation of *CCND3 *and *CCNE1 *mRNAs were specific for *miR-15a/16 *(Figure 6B and Additional file 2). In contrast, *CCNE2 *mRNA, which contains a target site for *miR-34a *[16], and *CCNA1 *mRNA, which contains no target site, were virtually unaffected.

**Figure 7:**
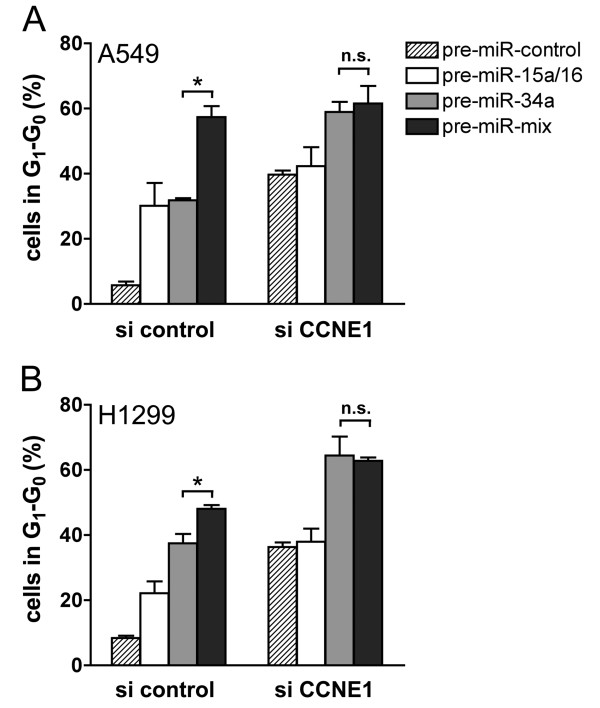
**Synergistic action on cell cycle arrest is due to the down-regulation of unique mRNA targets**. A549 (A) or H1299 cells (B) were co-transfected with 20 nM miRNA precursors and 7.8 nM siRNA against CCNE1 and subsequently treated for 18 h with nocodazole beginning 24 h post-transfection. Comparable results were also obtained 48 h post-transfection (data not shown).

To address the possibility that the synergistic effect was due to an increased number of targets, *CCNE1*, a target unique to *miR-15a/16 *(Figure 6B and Additional file 2), was knocked down in A549 cells by RNA interference resulting in 80.3 ± 6.6% less *CCNE1 *mRNA. We would expect that the synergistic effect would be reduced under these conditions, given that one of the unique targets, *CCNE1*, has been removed. This was indeed the case. The percentage of cells in the G_1_-G_0 _phase increased to 36% on co-transfection with CCNE1 siRNA and pre-miR-control (Figure 7A, striped columns). However, *CCNE1 *siRNA had only a low impact on cell cycle arrest of cells overexpressing *miR-15a/16 *(Figure 7A, white columns), which can be explained by the fact that *CCNE1 *was already down-regulated by *miR-15a/16*. In contrast, the percentage of A549 cells in G_1_-G_0 _increased almost two-fold on co-transfection with *pre-miR-34a *and *CCNE1 *siRNA relative to cells co-transfected with *pre-miR-34a *and si control (Figure 7A, grey columns), suggesting that *CCNE1 *siRNA and *pre-miR-34a *act together to induce cell cycle arrest in a more efficient manner. Notably, *CCNE1 *siRNA in combination with *pre-miR-34a *(59.0 ± 3.1%; grey column) and CCNE1 siRNA in combination with both pre-miRNAs (61.6 ± 5.3%; black column; p = 0.5) induced a G_1_-G_0 _arrest with the same efficiency. Thus, the synergistic effect exerted by the combined action of *miR-15a/16 *and *miR-34a *was clearly abrogated. In contrast, si control in combination with both pre-miRNAs together gave rise to almost two times more cells in G_1_-G_0 _than si control in combination with *pre-miR-34a or pre-miR-15a/16, respectively *(p < 0.004). The observed effects were not cell-line-specific, since comparable results were obtained for A549 (Figure 7A) and H1299 cells (Figure 7B). In conclusion, the synergistic effect of *miR-15a/16 *and *miR-34a *is due to the fact that more miRNA targets are down-regulated by the combined action of both miRNAs.

## Discussion

Cell cycle progression critically depends on numerous regulatory processes which are often deregulated in cancer (reviewed by Evan et al. [28]). miRNAs contribute to the complexity of cell cycle control by interfering with a variety of different components of the cell cycle machinery allowing the coordinated regulation of gene expression at the post-transcriptional level (reviewed by Bueno et al. [29] and Carleton et al. [30]).

*miR-15a/16 *and *miR-34a *share overlapping functions. They both induce cell cycle arrest in G_1_-G_0 _and share common targets including *CCND1*, *CDK4 *and *CDK6*. In addition, the ability of either one of these miRNAs to induce cell cycle arrest in G_1_-G_0 _largely depends on the expression of Rb (Figure 3 and ref. [7]). Cyclin D in complexes with CDK4 or CDK6, and cyclin E in a complex with CDK2 regulate progression through the G_1_-S boundary of the cell cycle. These complexes phosphorylate and thereby prevent Rb from binding to E2F, which on release, drives cells from G_1 _to S phase (reviewed by Morgan et al. [31]). From these results we may conclude that functionally relevant targets of either type of miRNA must be upstream of Rb. These include CCNE1 and CCND3, which are unique to miR-15a/16, c-Myc and c-Met, which are unique to *miR-34a *and CCND1, CDK4 and CDK6, which are common to both miRNAs. With the exception of CDK4 and c-Myc, all these genes are confirmed targets of *miR-15a/16 *and *miR-34a *in NSCLC cells [7,14,16,26]. In contrast, experimentally validated targets downstream of Rb including E2F1, E2F2, E2F3, E2F7, WEE1, CHK1 and CARD10 [25,32,33] seem to be less relevant for the regulation of cell cycle progression by *miR-15a/16 *or *miR-34a*, at least in NSCLC cells.

The finding that both miRNAs share highly related functions is further illustrated by the fact that both miRNAs are co-regulated in all adenocarcinoma samples. In the majority of NSCLC cases, both miRNAs are significantly down-regulated indicating that they play an important role as tumour suppressor. Tumours can escape the concerted action of *miR-15a/16 *and *miR-34a *by down-regulation of both miRNAs or, alternatively, by down-regulation of Rb. Mechanisms which may lead to dysregulation of *miR-15a/16 *or *miR-34a *in cancer include deletion of the respective miRNA loci [7,34], defects in miRNA processing [21], altered promoter methylation [35], or altered expression of transcriptional regulators [36-38]. Defects in miRNA processing may account for only a subgroup of NSCLC, since the majority of tumours either expressed normal or high levels of miR-21. p53 is a potent transactivator of *miR-34a *[39,40], and is implicated in the processing of *miR-16 *[40]. However, no correlation was observed between the mutation status of p53 and the expression level of miR-34a [6] or *miR-15a/16 *[41]. In addition, the possibility that both miRNAs are able to mutually regulate their expression can be excluded (Figure 1C). Thus, it rather seems that several independent mechanisms may account for the dysregulation of *miR-15a/16 *and *miR-34a *in NSCLC.

Why is there a relatively high degree of redundancy between miRNAs? To address this question we co-transfected cells with *miR-15a/16 *and *miR-34a *and demonstrated that both miRNAs act synergistically to induce cell cycle arrest in G_1_-G_0_. In contrast, the concerted action of these miRNAs on common mRNA targets was additive rather than synergistic. Thus, there seems to be little interference in binding of these miRNAs to the same target molecule and each miRNA contributes to the mRNA stability in an independent manner. The synergistic effect can rather be explained by the fact that in addition to their targets common to both miRNAs they are also able to bind to targets unique to either type of miRNA. Thus, in a combinatorial mode, both miRNAs are able to down-regulate more targets than each miRNA alone. This is based on the finding that knocking down *CCNE1*, a target unique to *miR-15a/16*, by RNA interference, abrogated the synergistic effect exerted by the combination of both miRNAs (Figure 7). These effects were not cell-line-specific, since comparable results were obtained with A549 and H1299 cells. This model is in agreement with our results that *miR-34a *and *miR-15a/16 *acted synergistically under both saturating and non-saturating conditions. In contrast, if the synergistic effect of these miRNAs were due to a more efficient repression of individual targets, we would expect such an effect to occur only under non-saturating conditions. miRNAs exert fine-tuning regulatory functions, in most cases leading only to a modest repression of target mRNAs and proteins [24]. Our results suggest that miRNAs can potentiate their impact on the regulation of cellular processes by acting in a combinatorial mode.

Surprisingly, we were unable to detect any synergistic effect on apoptosis. Although both miRNAs are able to target *Bcl2*, only *miR-34a *was able to induce apoptosis. This may be due to quantitative differences in their ability to down-regulate *Bcl2*. Alternatively, other anti-apoptotic genes besides *Bcl2*, which are targeted by *miR-34a*, but not *miR-15a/16*, may have to be down-regulated in order apoptosis can occur. It is noteworthy, however, that the observed effects may depend on the cell system as *miR-15a/16 *was able to induce apoptosis in CLL [15].

There are only few examples of miRNAs in the literature that act in a synergistic manner. Ivanosvska and Cleary were the first to investigate the concerted action of miR-16 and miR-34a on cell cycle arrest. However, based on their results it was not clear if both miRNAs acted in an additive or synergistic manner [42]. *miR-84 *and *let-7 *promote terminal differentiation of the hypodermis and cessation of molting in *C. elegans *in a synergistic manner [43]. However, *miR-84 *and *let-7 *share identical seed sequences, suggesting that they regulate the same set of target genes. In addition, pairs of a cytomegalovirus derived miRNA and a host cell derived miRNA acted on the same gene (*MICB*) through site proximity in a synergistic manner [44]. Thus different mechanisms may exist that may lead to a synergistic action of miRNAs.

Therapeutic strategies for the treatment of human cancer based on modulation of miRNA activity in cancer tissues have gained much attention in the past few years [12,45-49]. In a recent publication, a new formulation is described that allows the reintroduction of miRNAs, depleted in cancer cells, in order to reactivate cellular pathways that drive a therapeutic response [50]. The authors demonstrated that formulated *miR-34a *blocked tumour growth in a mouse model of NSCLC. Our results suggest that administering formulated *miR-34a *in combination with formulated *miR-15a/16 *may lead to a significant increase in the therapeutic impact. This strategy may be particularly effective for the treatment of NSCLC, since both types of miRNAs are normally down-regulated in this class of tumours.

## Conclusion

It is generally agreed that miRNAs form part of networks to control cellular processes. Currently, the miRNA field is focused primarily on the identification of novel targets of individual miRNAs, but little information is available how miRNAs act in a combinatorial mode. We show that *miR-34a *and *miR-15a/16 *act together to control cell cycle progression in a synergistic and Rb-dependent manner. From these results we may conclude that the combination of miRNAs, which form part of the same network, rather than individual miRNAs should be considered for assessing a biological response. In addition, our study may have translational implications. Since both miRNAs are significantly down-regulated in the majority of adenocarcinomas, administering a combination of both miRNAs may potentiate the therapeutic impact of each individual miRNA.

## Competing interests

The authors declare that they have no competing interests.

## Authors' contributions

NB performed all experiments, participated in the conception and design of the study and helped to draft the manuscript. EV was responsible for the conception and design of the study and wrote the manuscript. Both authors read and approved the final manuscript.

## Supplementary Material

Additional file 1 **Analysis of propidium iodide (PI)-stained cells by flow cytometry**. H2009 and A549 cells were transfected as described in the legends to Figure 5 and analysed 72 h or 96 h post-transfection, respectively. (A) dot plot of FSC vs. PI (log) of the transfection experiments in Figure 5B. (B) percent PI-positive cells. The mean ± SD from independent transfections is presented (n ≥ 3). Click here for file

Additional file 2 **Schematic depiction of the 3' untranslated region of *miR-15a/16 *and *miR-34a *targets**. *miR-15a/16-*specific target sites are highlighted in red and *miR-34a*-specific target sites are highlighted in blue. *CCND1, CCND2, CCND3, CCNE1, CDK4, c-MET *and *Bcl2 *are experimentally validated targets and *CDK6 *and *c-MYC *are predicted targets of *miR-15a/16 *and *miR-34a *in NSCLC cell lines. CCNA1 contains no *miR-15a/16 *or *miR-34a*-specific target sites. Click here for file
