# Supplementary figures and images for: miR-34a and miR-15a/16 are co-regulated in non-small cell lung cancer and control cell cycle progression in a synergistic and Rb-dependent manner
Source: Mol Cancer. 2011 May 16;10:55. doi: 10.1186/1476-4598-10-55 (PMC3120797; doi:10.1186/1476-4598-10-55)

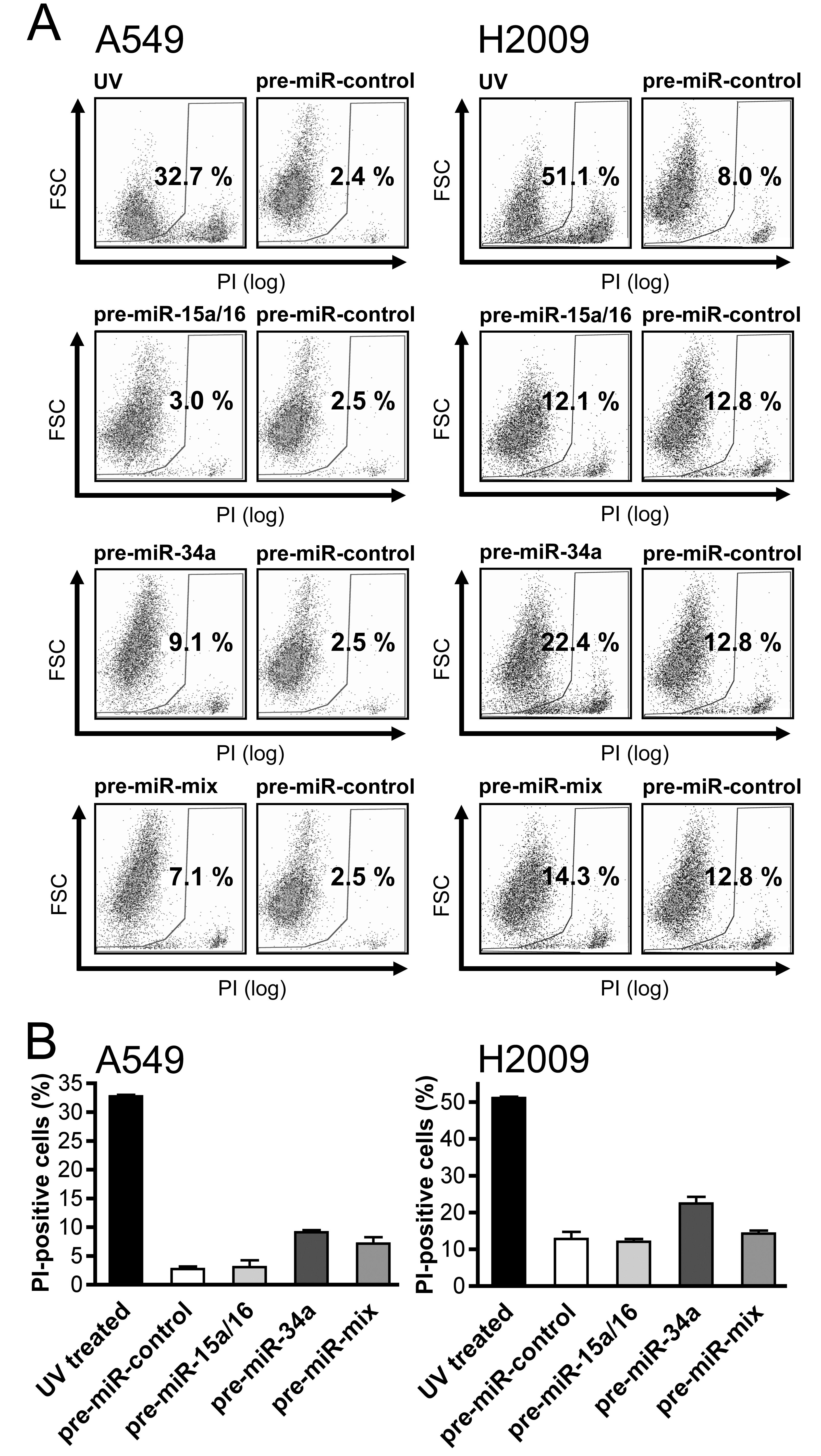

Supplement: Additional file 1 — Analysis of propidium iodide (PI)-stained cells by flow cytometry. H2009 and A549 cells were transfected as described in the legends to Figure 5 and analysed 72 h or 96 h post-transfection, respectively. (A) dot plot of FSC vs. PI (log) of the transfection experiments in Figure 5B. (B) percent PI-positive cells. The mean ± SD from independent transfections is presented (n ≥ 3). [file 1476-4598-10-55-S1.TIFF]

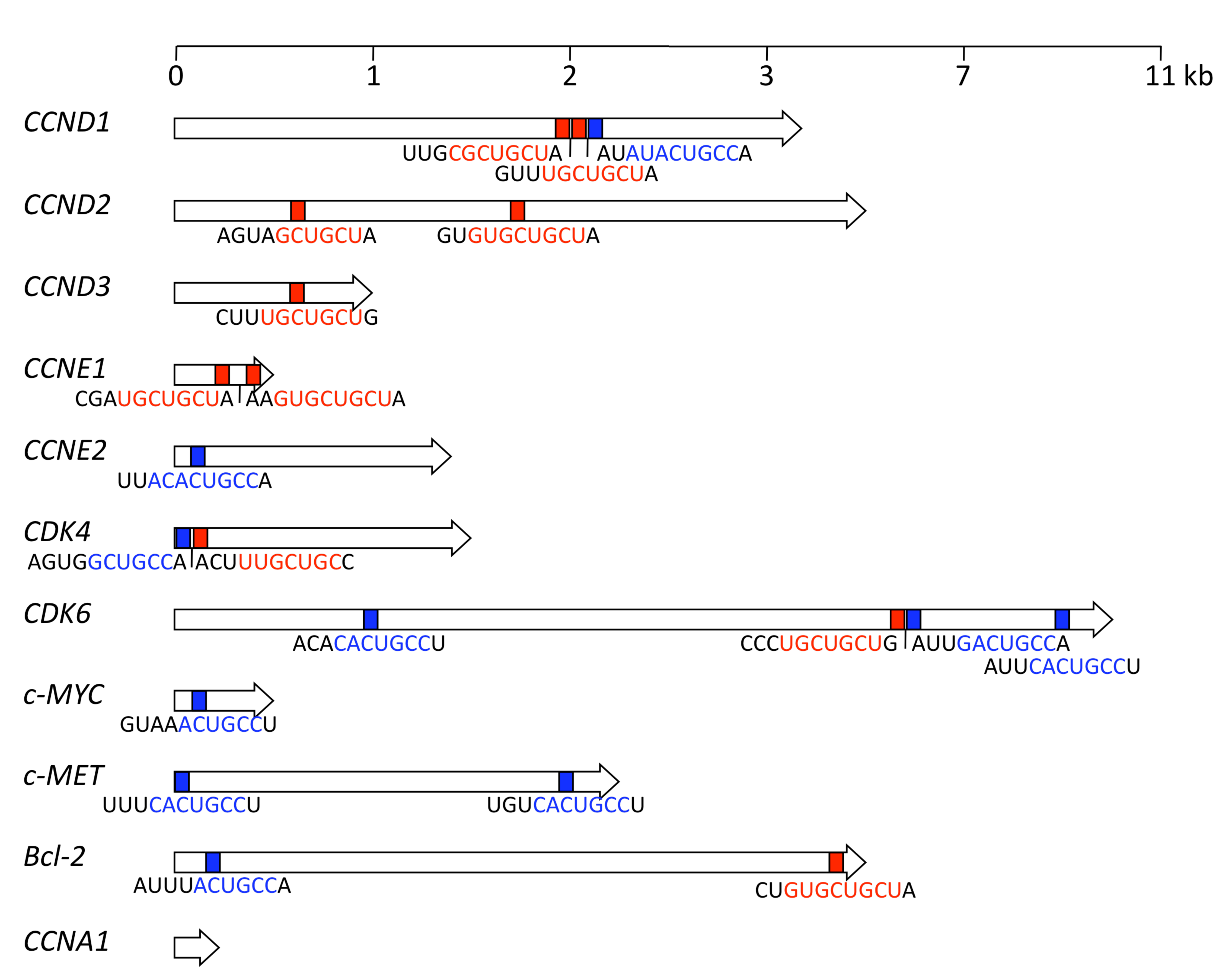

Supplement: Additional file 2 — Schematic depiction of the 3' untranslated region of miR-15a/16 and miR-34a targets. miR-15a/16-specific target sites are highlighted in red and miR-34a-specific target sites are highlighted in blue. CCND1, CCND2, CCND3, CCNE1, CDK4, c-MET and Bcl2 are experimentally validated targets and CDK6 and c-MYC are predicted targets of miR-15a/16 and miR-34a in NSCLC cell lines. CCNA1 contains no miR-15a/16 or miR-34a-specific target sites. [file 1476-4598-10-55-S2.TIFF]
